# Detection of a novel stem cell probably involved in normal turnover of the lung airway epithelium

**DOI:** 10.1111/jcmm.12653

**Published:** 2015-08-10

**Authors:** Marta Ortega-Martínez, Laura E Rodríguez-Flores, Carlos de-la-Garza-González, Jesús Ancer-Rodríguez, Gilberto Jaramillo-Rangel

**Affiliations:** a Department of Pathology, School of Medicine, Autonomous University of Nuevo LeonMonterrey, Nuevo Leon, Mexico; b Department of Embryology, School of Medicine, Autonomous University of Nuevo LeonMonterrey, Nuevo Leon, Mexico

**Keywords:** lung airway epithelium, stem cell, nestin

## Abstract

Regeneration of the lung airway epithelium after injury has been extensively studied. In contrast, analysis of its turnover in healthy adulthood has received little attention. In the classical view, this epithelium is maintained in the steady-state by the infrequent proliferation of basal or Clara cells. The intermediate filament protein nestin was initially identified as a marker for neural stem cells, but its expression has also been detected in other stem cells. Lungs from CD1 mice at the age of 2, 6, 12, 18 or 24 months were fixed in neutral-buffered formalin and paraffin-embedded. Nestin expression was examined by an immunohistochemical peroxidase-based method. Nestin-positive cells were detected in perivascular areas and in connective tissue that were in close proximity of the airway epithelium. Also, nestin-positive cells were found among the cells lining the airway epithelium. These findings **suggest** that nestin-positive stem cells circulate in the bloodstream, transmigrate through blood vessels and localize in the lung airway epithelium to participate in its turnover. We previously reported the existence of similar cells able to differentiate into lung chondrocytes. Thus, the stem cell reported here **might be** a bone marrow-derived mesenchymal stem cell (BMDMSC) able to generate several types of lung tissues. In conclusion, our findings indicate that there exist a BMDMSC in healthy adulthood that participates in the turnover of the lung airway epithelium. These findings may improve our knowledge about the lung stem cell biology and also provide novel approaches to therapy for devastating pulmonary diseases.

## Introduction

The airway epithelium forms a selectively permeable layer that serves as a first-line barrier of defence against exogenous agents. It includes stem/progenitor cells, which can proliferate in response to injury, leading to self-renewal or to the generation of other specialized epithelial cell types 1. Classically, the lung airway epithelium has been divided into regions with their own stem/progenitor cells which can repopulate the tissue. Thus, the local repopulating cells of the bronchus (basal) and bronchiole (Clara) remain as the first reserve of stem/progenitor cells 2,3.

Regeneration of the lung airway epithelium after injury because of several factors has been extensively studied 4,5. In contrast, analysis of its turnover in healthy adulthood has received little attention. In the classical view, this epithelium is maintained in the steady-state by the infrequent proliferation of basal or Clara cells 6,7.

The search of a pluripotent cell giving rise to any lung tissue has not yet been successful and the role of circulating bone marrow stem cells in the lung tissue renewal has not been completely elucidated 2,8,9.

We have previously reported the existence of nestin-positive stem cells in the healthy adult mouse able to differentiate into lung chondrocytes. They might be circulating in the bloodstream and be able to populate the cartilage 10,11. Here, we present evidence that those cells might also participate in the turnover of the lung airway epithelium.

## Materials and methods

Animals and experimental procedures used were described in two previous papers 10,11. Briefly, male CD1 mice were examined; they were kept in standard conditions: stainless-steel cages, receiving chow and water *ad libitum*, 18–21°C, 55–60% relative humidity, and 12:12 hr day-night cycle. Three animals were killed at the age of 2, 6, 12, 18 or 24 months. Right lungs were fixed in 10% neutral-buffered formalin and paraffin-embedded. Serial 5-μm sections were cut, deparaffinized in xylene and hydrated in a graded series of alcohol. Sections were subjected to a heat-induced epitope retrieval step performed by microwave treatment at 89°C for 10 min. in 0.01 M citrate buffer (pH 6.0). Endogenous peroxidase activity was blocked with 3% hydrogen peroxide at room temperature for 15 min. The slides were incubated with a monoclonal antibody against nestin (1:100; Millipore, Billerica, MA, USA). Incubations were conducted overnight at room temperature in a humid chamber. Detection was carried out with the antimouse Ig HRP Detection Kit (BD Pharmingen, San Diego, CA, USA) used according to the manufacturer's instructions; diaminobenzidine was applied as chromogen. Slides were counterstained with 0.5% methyl green. In control experiments, the primary antibody incubation step was omitted. All work involving animals was undertaken in accordance with EU Directive 2010/63/EU for animal experiments (http://ec.europa.eu/environmental/chemicals/lab_animals/legislation_en.htm).

## Results and discussion

**Figure 1:**
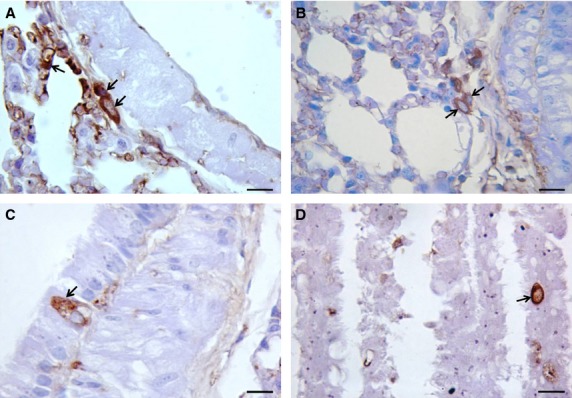
Detection of nestin-positive stem cells (blue nucleus/brown cytoplasm, arrows) in lung sections from healthy adult mice. Nestin-positive cells were detected in perivascular areas (**A**) and in connective tissue (**B**) that were in close proximity of the bronchial airway epithelium. Nestin-positive cells were also found among the cells lining the bronchial airway epithelium (**C** and **D**). Scale bar = 10 μm.

Between one and five nestin-positive cells were found in each lung specimen of mice at 2, 6, 12 and 18 months of age in perivascular areas (Fig.1A) and in connective tissue (Fig.1B) that were in close proximity of the bronchial airway epithelium. Also, nestin-positive cells were found among the cells lining the bronchial airway epithelium (Fig.1C and D).

Our findings **suggest** that nestin-positive stem cells circulate in the bloodstream, transmigrate through blood vessels, travel through the connective tissue, and ultimately localize in the epithelium to participate in its turnover. We previously reported the existence of a nestin-positive stem cell population similar to the described here, able to differentiate into lung chondrocytes in the healthy adult mouse 10,11. Bone marrow-derived mesenchymal stem cells (BMDMSCs) are a group of cells that have been shown capable of differentiating into a variety of cell types, including chondrocytes and epithelial cells 12. The intermediate filament protein nestin was initially identified as a marker for neural stem cells, but its expression has also been detected in BMDMSCs 13. Thus, the stem cell reported here **might be** a pluripotent cell able to generate several types of lung tissues. Cortiella *et al*. 14 and Vacanti *et al*. 15 presented evidence that a pluripotent stem cell exists in the lungs of adult sheep and rats that can generate lung-like tissue *in vitro*. Similar cells were found throughout the body, in each case giving rise *in vitro* to the tissue from which they were isolated 8,15.

The **possibility** that circulating BMDMSCs participate in the normal turnover of the lung airway epithelium is further supported by observations obtained in mouse lung injury models. Rojas *et al*. found that myelosupression increased susceptibility of lung to bleomycin injury and that BMDMSCs transfer was protective. Protection was associated with the differentiation of engrafted BMDMSCs into specific and distinct lung cell phenotypes 12. Also, Wong *et al*. identified a bone marrow-derived cell population capable of repopulate naphthalene-injured mouse airway epithelium. Those cells preferentially homed to naphthalene-damaged airways when delivered transtracheally or intravenously 9.

The identification of new types of stem cells over the past few years has led to models redefining the development of tissues and the lineage relationships that exist between adult cells 16,17. Turnover of the lung airway epithelium in healthy adulthood has been classically attributed to basal and Clara cells. Here, we present evidence that a new type of stem cell **might be** also involved in this process. Airway epithelial cells play a central role in the pathogenesis of chronic lung diseases, including chronic obstructive pulmonary disease, asthma and cystic fibrosis 18. The identification of new stem cells could provide new therapeutic opportunities for these pulmonary diseases by providing means to repair the lung and a novel route for gene therapy.

In conclusion, our findings indicate that there exist BMDMSCs positives for nestin in healthy adulthood that participate in the turnover of the lung airway epithelium. These findings may improve our knowledge about the lung stem cell biology and may also provide novel approaches to therapy for devastating pulmonary diseases. More research is necessary to determine the exact origin of these cells and factors regulating their function.

## Conflicts of interest

The authors confirm that there are no conflicts of interest.

## Supporting Information

**Figure S1** Histological sections stained with hematoxylin and eosin to show more precisely the localization of the nestin-positive cells showed in the Figure 1 of the manuscript.

**Figure S2** Isotype control was carried out on serial lung sections taken from the same specimens showed in Figure 1 of the manuscript (**A** in this figure corresponds to A in Fig. 1 and so on).
