# Supplementary material for: Detection of a novel stem cell probably involved in normal turnover of the lung airway epithelium
Source: J Cell Mol Med. 2015 Aug 10;19(11):2679–81. doi: 10.1111/jcmm.12653 (PMC4627572; doi:10.1111/jcmm.12653)
Supplement: Supplementary file 1 — Figure S1 Histological sections stained with hematoxylin and eosin to show more precisely the localization of the nestin-positive cells showed in the Figure 1 of the manuscript. [file jcmm0019-2679-sd1.docx]

Histological sections stained with hematoxylin and eosin to show more precisely the localization of the nestin-positive cells showed in the Figure 1A of the manuscript. Asterisk: area where the nestin-positive cells were found; BV: blood vessel; Arrowhead: bronchial airway epithelium; Dotted arrow: alveoli. Left: 100x; Right: 400x (images of the manuscript were at 1000x).

Histological sections stained with hematoxylin and eosin to show more precisely the localization of the nestin-positive cells showed in the Figure 1B of the manuscript. Circle and Asterisk: area where the nestin-positive cells were found; Arrowhead: bronchial airway epithelium; Dotted arrow: alveoli. Left: 100x; Right: 400x (images of the manuscript were at 1000x).

Histological sections stained with hematoxylin and eosin to show more precisely the localization of the nestin-positive cells showed in the Figure 1C of the manuscript.

Left. Circle: area where the nestin-positive cells were found; Arrowhead: bronchial airway epithelium; Dotted arrow: alveoli; Arrow: cartilage.

Right. Arrowhead: area where the nestin-positive cells were found; SM: smooth muscle.

Left: 100x; Right: 400x (images of the manuscript were at 1000x).

Histological sections stained with hematoxylin and eosin to show more precisely the localization of the nestin-positive cells showed in the Figure 1D of the manuscript. Circle: area where the nestin-positive cells were found; Arrow: cartilage; Dotted arrow: alveoli. Left: 100x; Right: 400x (images of the manuscript were at 1000x).
