# Supplementary material for: Detection of a novel stem cell probably involved in normal turnover of the lung airway epithelium
Source: J Cell Mol Med. 2015 Aug 10;19(11):2679–81. doi: 10.1111/jcmm.12653 (PMC4627572; doi:10.1111/jcmm.12653)
Supplement: Supplementary file 2 — Figure S2 Isotype control was carried out on serial lung sections taken from the same specimens showed in Figure 1 of the manuscript (A in this figure corresponds to A in Fig. 1 and so on). [file jcmm0019-2679-sd2.docx]

Isotype control was carried out on serial lung sections taken from the same specimens showed in Figure 1 of the manuscript (A in this figure corresponds to A in Figure 1 and so on). Magnification in this figure and Figure 1 is the same.

An isotype control is an antibody of the same isotype that the primary antibody with no relevant specificity to the target antigen. Furthermore, host specie and protein concentrations used in assays must be equal for both antibodies.

The characteristics of the primary antibody and of the isotype control used were:

|  | **Primary antibody** | **Isotype control** |
| --- | --- | --- |
| Name | Mouse anti-nestin monoclonal antibody | Mouse anti-neuronal nuclei (NeuN) monoclonal antibody |
| Source | Millipore | Millipore |
| Catalog number | MAB353 | MAB377 |
| Specificity | Rodent nestin protein confirmed with rat and mouse | Vertebrate neuron-specific nuclear protein called NeuN (Neuronal Nuclei) |
| Isotype | IgG_1_ | IgG_1_ |
| Host | Mouse | Mouse |
| Concentration | 1.02 mg/mL | 1 mg/mL |

Experimental procedures used with primary antibody and isotype control were identical and are described in the manuscript.

No signal was detected in the isotype control.

Detection of the proliferating cell nuclear antigen (PCNA) was carried out on serial lung sections taken from the same specimens showed in Figure 1 of the manuscript (A in this figure corresponds to A in Figure 1 and so on). Magnification in this figure and Figure 1 is the same.

In contrast to the cytoplasmic signal observed in the detection of nestin, note the nuclear reaction obtained in the detection of PCNA. Thus, we can detect clear and unambigously different antigens in the same specimen. This is another evidence that the signal observed in the Figure 1 of our manuscript is specific to nestin.

Since it is known that kidney shows a positive reaction for nestin in glomerulus [1, 2], we used it as a positive control (A). In B the negative control of this reaction is shown. Magnification: 400x.

1. **Chen J, Boyle S, Zhao M, *et al*.** Differential expression of the intermediate filament protein nestin during renal development and its localization in adult podocytes. *J Am Soc Nephrol*. 2006; 17: 1283-91.
2. **Bertelli E, Regoli M, Fonzi L, *et al*.** Nestin expression in adult and developing human kidney. *J Histochem Cytochem*. 2007; 55: 411-21.
